# Book Reviews

**Published:** 1897-01

**Authors:** 


A Treatise of Appendicitis. By John B. Deaver, M. D., Surgeon to the German Hospital, Philadelphia. Containing thirty-two full-page plates and other illustrations. Philadelphia: P. Blakiston, Son & Co., 1012 Walnut street. 1896.

The rapidly increasing literature of appendicitis has lately been still further enriched by the work of Dr. Deaver now under consideration. The author states that he has been prompted to write this book by the belief that the importance of this disease entitles it to more thorough and exhaustive study than has heretofore been accorded it. It is well known that it is a disease capable of manifold symptoms and serious complications, demanding much versatility on the part of the surgeon, who must not only be familiar with its usual forms in which diagnosis is comparatively easy, but he must also be prepared to meet its varied and divers manifestations and its multiform complications.

Deaver states that he has endeavored to emphasise the etiology, symptomatology, and special technique in the operative treatment of appendicitis in this work, and that the observations contained in it are the results of an experience in the treatment of over 500 cases of the disease. Surely such a wide experience ought to enable a man to speak from the chair. He arranges the subject into the following divisions: History, anatomy, etiology, pathology, symptoms, diagnosis, differential diagnosis, prognosis, treatment, complications, sequelæ and after-treatment. It is a well-formulated plan, and considers the subject with heads arranged in a logical order of sequence. It is illustrated in an admirable manner, many of the plates being executed in colors, and their clearness serves to delineate many obscure points that have not heretofore been attempted by figures or drawings. These, for the most part, are taken from cases, and this is uniformly the case where pathological demonstrations are made. One of the most useful parts that the illustrations serve is that connected with the operation itself. Never before has the surgical anatomy of the incision been so admirably shown by cuts, nor have the various conditions of the appendix *in situ* been so beautifully exhibited through the incision by artistic reproductions.

The book is printed on heavy paper in large modern-faced type, and its entire mechanical execution is beyond criticism. While we do not anticipate that this treatise will solve the entire problem of diagnosis and operative treatment of appendicitis, nor entirely fill the yawning gap that exists between a classic case and one of extreme complications, yet we do believe it will go a long way toward accomplishing its purpose in this regard, and, moreover, that it is a decided contribution to the literature of an essentially surgical disease, full of helpful hints and containing many novel suggestions that are useful in character.

---

THE PRACTICE OF MEDICINE. By HORATIO C. WOOD, A. M., M. D., Professor of Therapeutics and Clinical Professor of Nervous Diseases in the University of Pennsylvania, etc., and REGINALD H. FITZ, A. M., M. D., Hersey Professor of the Theory and Practice of Physic in Harvard University, etc. Octavo, pp. x.—1088 Philadelphia: J. B. Lippincott Co. London: 10 Henrietta street, Covent Garden. 1896.

No matter how many other treatises on the practice of medicine already exist, the fact that these two authors have combined in writing a new one will be welcome news to the medical profession. Since the work was announced it has been eagerly awaited by all who are familiar with the previous teachings and writings of the authors. Dr. Wood has made himself familiar to physicians all over the world in his chosen field of labor, as well as through his appearance on the rostrum at commencement and other anniversary occasions. Dr. Fitz is rapidly becoming as famous, and whenever he publishes an article it is eagerly read by a vast number of physicians throughout the country. We, therefore, feel sure that the treatise under consideration will not suffer for want of purchasers.

This work is divided into six sections. The first, devoted to general diseases, consists of five chapters, in which diseases of the blood and of the ductless glands, locomotor and constitutional diseases, infectious diseases, diseases due to animal parasites and poisonings are considered. In the second section, allotted to the consideration of diseases of the nervous system, we note seven chapters, divided as follows: general symptomatology; functional and nervous diseases; organic diseases of the brain and its membranes; diseases of the medulla oblongata; organic diseases of the spinal cord and its membranes; organic diseases of the nerves, vaso-motor and trophic diseases. In section third, assigned to the consideration of diseases of the circulatory apparatus, are four chapters that subdivide the subject as follows—namely : diseases of the pericardium ; diseases of the heart and myocardium ; diseases of the endocardium ; and diseases of the arteries. Section four is set apart to the consideration of diseases of the respiratory apparatus. Here the story is told in three chapters under the following heads : diseases of the nose, larynx, trachea and bronchi ; diseases of the lungs ; diseases of the pleura and the mediastinum. Section five presents diseases of the digestive apparatus and of the peritoneum in five chapters : diseases of the mouth, pharynx and esophagus ; diseases of the stomach ; diseases of the intestine ; diseases of the liver, gall-bladder and bile ducts ; diseases of the pancreas and of the peritoneum. In section six we find diseases of the urinary apparatus handled in two chapters : the first considers diseases of the kidneys ; the second, cysts and tumors of the kidneys, and diseases of the renal pelvis and the bladder.

If we should attempt an analysis in detail of this valuable treatise it would occupy space enough to weary our readers, to say nothing of the reviewer himself. We have thought we could best serve those in interest by giving the foregoing synopsis of the contents of the work, which will enable the searcher to determine the comprehensiveness with which it seeks to occupy the field and also indicate the method in which the authors have prepared this new candidate for favor.

In an appendix some useful formulæ will be found, as well as a number of temperature charts. The book closes with a comprehensive three-column index that makes its text unusually accessible. The paper on which the book is printed is somewhat inferior in quality, though the type and presswork are excellent. The style of the authors is so concise that much more than ordinary ground is covered without increasing the usual number of pages allotted to similar works.

The student and young practitioner of medicine will find it one of the best arranged treatises yet offered, and we shall be very much mistaken if older physicians do not find it of sufficient interest to become extensive purchasers.

---

Food in Health and Disease. By I. Burney Yeo, M. D., F. R. C. P., Professor of Therapeutics in King's College, London. New (second) edition. In one 12mo volume of 592 pages, with four engravings. Cloth, $2.50. Philadelphia and New York : Lea Brothers & Co., Publishers. 1896.

Since 1889, when the first edition of this work appeared, there have been several reprints in which no marked changes were made ; but in this edition it will be found that the author has endeavored to keep pace with advances and has brought it forward to embrace the conditions of the present. In doing so it has become necessary to make a number of changes and additions as well as to omit some paragraphs or portions that were regarded as approaching obsolescence.

There is no questioning the fact that food is one of the most important agents in the management of disease, because problems of nutrition lay at the foundation of by far the largest proportion of diseases to which the human body is liable. This fact alone would make this treatise valuable to physicians, but coupled with the fact that Yeo is especially competent to deal with the subject, makes it a book doubly important to possess.

One of the most important questions connected with the food in disease is the fixation of the proper diet during convalescence. After exhaustive diseases like typhoid fever there comes a time of special danger from improper as well as over feeding. These matters are often left to the judgment of incompetent persons, hence frequently lead to dangerous relapse and sometimes fatal results. If physicians would study into the relationship of food to disease more closely, and especially mark out a fixed and determined dietary for convalescence, there would be more prompt recovery from many prolonged or wasting diseases. From this work of Yeo may be gleaned information that may lead to such a consummation. We should be glad if every physician would study it carefully and follow its teachings with precision. The book is handsomely printed and bound, and is of a size convenient for reference.

---

A Manual of Materia Medica and Pharmacology. Comprising all organic and inorganic drugs which are and have been official in the United States Pharmacopeia, together with important allied species and useful synthetics. For students of medicine, druggists, pharmacists and physicians. By David M. R. Culbreth, M. D., Professor of Botany, Materia Medica and Pharmacognosy in the Maryland College of Pharmacy, Baltimore. In one octavo volume of 812 pages, with 445 illustrations. Philadelphia and New York: Lea Brothers & Co., Publishers. 1896.

This treatise is a valuable addition to the literature of the subject which it considers. The author is a teacher of distinction, and embodies in its pages an experience of ten years in class instruction as well as that of twenty years in a drug store. It would, therefore, appear that he is familiar with the necessities of modern pharmacists. That he has demonstrated this through the method that he has adopted in preparing this treatise is clearly apparent upon examining the text. His style is not the smoothest, but it is forceful. He presents his subject under four general heads—first, all official drugs, organic and inorganic, included in our pharmacopeia, together with preparations, official and nonofficial; second, the drugs once official in previous editions in our pharmacopeia, but now dropped; third, allied specimens of organic drugs; fourth, important unofficial synthetic compounds. He has added a valuable part, in which he considers the microscope and its use in materia medica. In an appendix, poisons, with their treatment and antidotes, are set forth. A comprehensive index concludes the volume. We commend the illustrations as being especially valuable ; their number in this work is unusual, and their excellence is apparent on inspection. We regard this as one of the best of the recent treatises on materia medica and pharmacology, and one that ought to interest especially the student of pharmacy.

---

Functional Disorders of the Nervous System in Women. By T. J. McGillicuddy, A. M., M. D., Consulting Physician to the Italian Hospital, New York, etc., etc. Small 8vo, pp. 367. Illustrated. New York : William Wood & Co. 1896.

This author has undertaken a departure from the usual course and has presented us a new treatise, or rather has given us thoughts and ideas in a new form. Instead of being obliged to search through the several treatises on diseases of women, diseases of the nervous system, etc., we can now find in a single volume grouped the most important functional disorders of the nervous system in women.

The largest part of this work is devoted to the consideration of the several neuroses—reflex, cerebral, spinal, cardiac, vascular, gastric, intestinal, vesical, etc., etc., not forgetting, to be sure, the hystero-neuroses, which is a general term for neurotic conditions of the genital system. Chapters are added on hysteria, hystero-epilepsy, hemicrania, or migraine, and therapeutics. The work is handsomely illustrated with colored plates and wood engravings. It contains a bibliographical index and an appendix embracing ten charts and diagrams, which serve to assist in explaining the text, and are especially useful, too, in assisting to reach a correct diagnosis. We consider this book a decided addition to the literature of the diseases of women and one that will be welcome to both gynecologist and neurologist.

---

A Practical Treatise on Medical Diagnosis. For the use of students and practitioners. By John H. Musser, M. D., Assistant Professor of Clinical Medicine, University of Pennsylvania, Philadelphia. New (second) edition, thoroughly revised. In one octavo volume of 925 pages, with 177 engravings and eleven full-page colored plates. Philadelphia and New York : Lea Brothers & Co. 1896.

That Musser's Diagnosis should have advanced to its second edition so soon is abundant proof of its popularity. The first edition was published in February, 1894, and became exhausted within less than two years. The importance of the subject with which this volume deals cannot be overestimated. Diagnosis is at the foundation of all successful treatment, and only a few authors have attempted this field. Da Costa has long been recognised as authority, and his treatise on diagnosis has been accepted as a text-book in most schools. With the appearance of Musser it became necessary to divide the honors, as the latter's admirable treatise was recognised at once to possess exceptional merit.

This second edition has been revised in detail, so that it may be regarded as the latest exponent of the most scientific methods of diagnosis. Its illustrations are numerous and of excellent quality, although in this department we feel there is still room for improvement. The photograph might be made to play a more important part in this field than it has yet done. This author, however, has made great advances over others in this line, as well as in many of the departments treated of in his book. We can easily understand how Musser's Diagnosis will become a necessity for students as well as those who have entered the field of practice.

---

A VEST-POCKET MEDICAL DICTIONARY. Embracing those terms and abbreviations which are commonly found in the medical literature of the day, but excluding the names of drugs and of many special anatomical terms. By ALBERT H. BUCK, M. D., New York City. New York: William Wood & Co. 1896.

For the special convenience of students this little dictionary, prepared by an experienced author, has been issued by the publishers. The aim has been to offer a dictionary containing a large number of new words which have been introduced during the past few years, and to record the significant changes that have taken place in some of the old terms.

A somewhat cursory examination of this dictionary discloses the fact that it is particularly rich in the most recent terms and newly created words. Röntgen rays are especially clearly defined at sufficient length to meet the requirements of the average searcher for concise information in the subject. This work aims to give those words which one is apt to meet in books or papers on the newer topics of medicine as well as the standard terms used in modern text-books, a task which it seems to have planned and satisfactorily fulfilled. It is one of the most convenient of the small medical dictionaries, and its binding in flexible red morocco is handsome and durable.

---

A MANUAL OF VENEREAL DISEASES. By JAMES R. HAYDEN, M. D., Chief of Venereal Clinic, College of Physicians and Surgeons, New York; Professor of Genito-urinary and Venereal Diseases in the Medical Department of the University of Vermont, etc. In one 12mo volume of 263 pages, with forty-seven engravings. Philadelphia and New York: Lea Brothers & Co. 1896.

The literature of late on venereal diseases has become somewhat voluminous. Nevertheless, it is a subject that invites study and investigation at the hands of the best minds. So much confusion has grown out of want of proper understanding of the subject, and especially with reference to the differentiation of its several forms, that a clear, concise and accurate statement like this work is to be welcomed. Hayden has had abundant opportunities to make himself familiar with his subject, and his manual discloses the fact that he methodised his work and studied it closely. Every point is presented with clearness and without superfluous language or unnecessary verbiage. In this manual will be found a clear and practical explanation and differentiation of the three venereal diseases—gonorrhea, chancroid and syphilis, together with their complications and sequelæ. We commend it to both students and practitioners as a valuable exposition of the subject.

---

THE READY REFERENCE HAND-BOOK OF DISEASES OF THE SKIN. By GEORGE THOMAS JACKSON, M. D., Professor of Dermatology, Woman's Medical College of the New York Infirmary and in the University of Vermont; Chief of Clinic and Instructor in Dermatology, College of Physicians and Surgeons, New York. New (second) edition. In one 12mo volume of 589 pages, with sixty-nine illustrations and a colored plate. Philadelphia: Lea Brothers & Co. 1896.

This is essentially a hand-book, as its title indicates. The first edition was well received and has become exhausted; hence the author avails himself of the opportunity created by the demand for a second edition to so completely revise his work as to bring it forward to the present period. New illustrations, too, have been added and the text has been markedly increased. It is a work adapted to the needs of students, general practitioners of medicine and specialists. Jackson is an author of recognised merit and his contributions to medical literature always command large and attentive audiences. We feel sure that this last one will prove no exception to the rule.

---

INDEX-CATALOGUE OF THE LIBRARY OF THE SURGEON-GENERAL'S OFFICE, UNITED STATES ARMY. Authors and Subjects. Second series, Volume I. A—Azzurri. Washington: Government Printing Office. 1896.

This volume includes 6,346 author-titles, representing 6,127 volumes and 6,327 pamphlets. It also contains 7,884 subject-titles of separate books and pamphlets and 30,384 titles of articles in periodicals. It is prepared under the direction of Deputy Surgeon-General D. L. Huntington, who has succeeded Dr. John S. Billings in charge of the work.

---

REPORT OF THE COMMISSIONER OF EDUCATION FOR THE YEAR 1893-94. Volumes I. and II., pp. 2290. Washington: Government Printing Office. 1896.

As time advances, the reports emanating from the department of education become of constantly increasing interest. Public educators can scarcely get along without these annual issues as works of reference, and libraries should assuredly possess them.

The list of medical societies published on page 1632, *et seq.*, is quite defective and should be made complete in order to be valuable for reference. There is, however, much in this report of interest to the medical educator, and especially to those interested in advancing the standard of medical teaching.

